# Supplementary material for: Fe–Ce Bimetallic MOFs for Water Environment Remediation: Efficient Removal of Fluoride and Phosphate
Source: Nanomaterials (Basel). 2025 Oct 24;15(21):1623. doi: 10.3390/nano15211623 (PMC12610324; doi:10.3390/nano15211623)
Supplement: Supplementary file 1 [file nanomaterials-15-01623-s001.zip › nanomaterials-3923183-supplementary.pdf]

Supplementary Materials

Fe–Ce Bimetallic MOFs for Water Environment Remediation: Efficient Removal of Fluoride and Phosphate

Jinyun Zhao [1], Yuhuan Su [1], Jiangyan Song [2], Ruilai Liu [1], Fangfang Wu [1], Jing Xu [1,*], Tao Xu [1], Jilin Mu [1], Hao Lin [1,*] and Jiapeng Hu [1,3,*]

[1] Fujian Provincial Bamboo Engineering Technology Research Center, Wuyishan 354300, China; biaoduo@163.com (J.Z.); suyuhuan0001@163.com (Y.S.); wyulrl@163.com (R.L.); wuff2018@wuyiu.edu.cn (F.W.); Xulao@163.com (T.X.); mujilin@163.com (J.M.)
[2] College of Environmental Science and Engineering, Tongji University, Shanghai 200092, China; sjya@tongji.edu.cn
[3] College of Environment and Safety Engineering, Fuzhou University, Fuzhou 350001, China
* Correspondence: jingxu@wuyiu.edu.cn (J.X.); linhaosg@wuyiu.edu.cn (H.L.); wyuwqhjp@163.com (J.H.)

S1. Chemical Reagents

The reagents used in this study included ferric chloride (analytical grade, Sinopharm Chemical Reagent Co., Ltd.), cerium nitrate (analytical grade, Shanghai Macklin Biochemical Technology Co., Ltd.), terephthalic acid (98%, Shanghai Macklin Biochemical Technology Co., Ltd.), hydrochloric acid (36–38%, Sinopharm Chemical Reagent Co., Ltd.), N,N-dimethylformamide (DMF, analytical grade, Sinopharm Chemical Reagent Co., Ltd.), sodium hydroxide, absolute ethanol, and formic acid (analytical grade, Sinopharm Chemical Reagent Co., Ltd.). All other reagents were of analytical grade and used as received without further purification.

S2. Adsorption Experiments of Fluoride and Phosphate

A total of 0.01 g of Fe-Ce-MOF adsorbent was added to 50 mL solutions containing initial fluoride or phosphate concentrations of 20, 25, 30, 35, 40, 45, and 50 mg L^{-1}, respectively. The mixtures were shaken for 12 h and then filtered through a microporous membrane.

The fluoride concentration was determined as follows: 10 mL of the supernatant was transferred into a 50 mL volumetric flask, followed by the addition of 10 mL buffer solution and dilution to the mark with deionized water. The fluoride concentration was then measured using the potentiometric method[1, 2].

The phosphate concentration was determined as follows: a certain volume of filtrate was mixed with 25 mL deionized water, 2 mL ammonium molybdate, and 3 mL ascorbic acid in a 50 mL volumetric flask. The solution was diluted to the mark with deionized water, incubated in a water bath at 30 °C for 30 min, and the concentration was determined at 880 nm using a UV–Vis spectrophotometer[3, 4].

The adsorption capacity was calculated using Equation (1), and the removal efficiency was calculated using Equation (2):

$$q_e = \frac{v(C_0 - C_e)}{m} \tag{S1}$$

$$\eta\% = \frac{C_0 - C_e}{C_0} \times 100\% \tag{S2}$$

where q_e (mg g^{-1}): the equilibrium adsorption capacity; V (L): the volume of the initial solution; C_0 and C_e (mg L^{-1}): the initial and equilibrium concentrations, respectively; m (g): the mass of the adsorbent dosage; η (%): removal efficiency.

This study also investigated the effect of different pH conditions on the removal efficiency of fluoride and phosphate by the Fe-Ce-MOF adsorbent. The solution pH was adjusted using 0.1 mol L^{-1} NaOH or HCl to evaluate the influence of pH on the stability and adsorption performance of the adsorbent. In the batch equilibrium experiments, the effects of coexisting anions, including Cl^-, NO_3^-, SO_4^{2-}, CO_3^{2-}, HCO_3^-, PO_4^{3-}, and F^-, in the concentration range of 10–100 mg L^{-1} on the removal of fluoride and phosphate were also examined.

For the adsorption thermodynamics study, the adsorption efficiency of Fe-Ce-MOFs for fluoride and phosphate was evaluated at 25, 35, and 45 °C. Unless otherwise specified, the experimental conditions were as follows: adsorbent dosage of 0.01 g, initial fluoride and phosphate concentration of 20 mg L^{-1}, solution pH of 4, and adsorption time of 12 h.

Regeneration experiments of Fe-Ce-MOF adsorbent: The saturated Fe-Ce-MOF adsorbent after adsorption was collected and transferred into a 50 mL centrifuge tube, followed by the addition of 30 mL of 0.1 mol L^{-1} NaOH solution. The mixture was shaken at 150 rpm for 2 h in a thermostatic shaker to achieve the desorption of fluoride or phosphate ions. After desorption, the adsorbent was separated by centrifugation, and the supernatant was discarded. The solid was washed three times with deionized water until the washing solution reached a nearly neutral pH. The washed adsorbent was then dried in

a vacuum oven at 50 °C for 12 h to obtain the regenerated Fe-Ce-MOFs. The above procedure was defined as one regeneration cycle, and the process was repeated for subsequent cycles (i.e., two times, three times, and so on).

In the treatment of real fluoride-containing wastewater, the field wastewater was collected from Shaowu Industrial Park, Fujian Province, China, and originated from equipment cleaning processes. For the treatment of real phosphorus-containing wastewater, the field wastewater was obtained from effluent generated during equipment cleaning at a fertilizer factory in Shunchang, Fujian Province, China.

S3. Characterization

The morphology and composition of adsorbent were determined by scanning electron microscopy hyphenated with energy dispersive X-ray spectroscopy (SEM-EDX) using equipment from Hitachi. The powder X-ray diffraction (XRD) pattern was recorded using a Bruker D8 ADVANCE with CuK_α to examine the crystal structure of the adsorbent, where the specific angle was selected from 5 to 70° and the scanning speed was selected at 10 °/min. N_2 sorption isotherms were measured at −196 °C using a Micromeritics Instrument (ASAP 2420), and the specific surface area was estimated by the Brunauer−Emmett−Teller (BET) method. The thermal stability of the adsorbent was studied by a thermogravimetric analysis (TGA) instrument (DTA-60H, DTG, SHIMADZU, Japan), in the temperature range from 20 to 800 °C under N_2 flow. The surface property of the adsorbent was examined using FTIR (Perkin Elmer) in the range of 400−4000 cm^{-1} using the KBr disk method. The elemental compositions and chemical valences were determined by XPS (ESCALAB250, Thermo VG, USA).

S4. Adsorption thermodynamics

The experimental data were fitted using the Langmuir and Freundlich isotherm models, expressed as follows[5, 6]:

Langmuir model:

$$\frac{C_e}{q_e} = \frac{1}{K_L q_m} + \frac{C_e}{q_m} \qquad \text{(S3)}$$

Freundlich model:

$$\lg q_e = \lg K_f + \frac{1}{n}\lg C_e \quad (S4)$$

where q_e (mg g^{-1}) is the equilibrium adsorption capacity, q_m (mg g^{-1}) is the maximum adsorption capacity, C_e (mg L^{-1}) is the equilibrium concentration, b (L mg^{-1}) is the Langmuir constant, K_f (mg g^{-1}) is the Freundlich adsorption constant, and n represents adsorption intensity.

To further clarify the adsorption mechanism, thermodynamic parameters including Gibbs free energy change (ΔG^0), enthalpy change (ΔH^0), and entropy change (ΔS^0) were calculated using the following equations[5, 7]:

$$K_D = \frac{q_e}{C_e} \quad (S5)$$

$$\Delta G^0 = -RT\ln K_D \quad (S6)$$

$$\ln K_D = \frac{\Delta S^0}{R} - \frac{\Delta H^0}{RT} \quad (S7)$$

where K_D is the distribution coefficient, R is the universal gas constant (8.314 J mol^{-1} K^{-1}), and T is the absolute temperature (K).

S5. Adsorption kinetics

Adsorption kinetics were analyzed by fitting the experimental data with pseudo-first-order, pseudo-second-order, and intraparticle diffusion models to elucidate the adsorption mechanism. Adsorption kinetics mainly describe the dynamic behavior of adsorbates on the adsorbent surface during the adsorption process, including the adsorption rate, influencing factors, and underlying mechanisms. In this study, the kinetic data were fitted using the pseudo-first-order, pseudo-second-order, and intraparticle diffusion models. The corresponding equations are expressed as follows [5, 6]:

Pseudo-first-order model:

$$\ln(q_e - q_t) = \ln q_e - k_1 t \quad (S8)$$

Pseudo-second-order model:

$$\frac{t}{q_t} = \frac{1}{k_2 q_e^2} + \frac{t}{q_e} \quad (S9)$$

Intraparticle diffusion model:

$$q_t = k_p t^{0.5} + C \quad (S10)$$

where q_t (mg g^{-1}) is the adsorption capacity at time t (min), qe (mg g^{-1}) is the equilibrium adsorption capacity, k_1 (min^{-1}) is the pseudo-first-order rate constant, k_2 (g mg^{-1} min^{-1}) is the pseudo-second-order rate constant, k_p (mg g^{-1} min$^{-1/2}$) is the intraparticle diffusion rate constant, and C is a constant.

References

[1] Liu R, Song J, Zhao J, Wang Z, Xu J, Yang W, Hu J, Novel MOF (Zr)–on-MOF (Ce/La) adsorbent for efficient fluoride and phosphate removal[J]. Chemical Engineering Journal, 2024, 497: 154780.

[2] Song J, Yang W, Han X, Jiang S, Zhang C, Pan W, Jian S, Hu J J M, Performance of rod-shaped Ce metal–organic frameworks for defluoridation[J]. Molecules, 2023, 28(8): 3492.

[3] Zhang L, Mao D, Qu Y, Chen X, Zhang J, Huang M, Wang J, Facile Synthesis of Ce-MOF for the Removal of Phosphate, Fluoride, and Arsenic[J]. Nanomaterials, 2023, 13(23): 3048.

[4] Nadagouda M N, Varshney G, Varshney V, Hejase C, Recent advances in technologies for phosphate removal and recovery: a review[J]. ACS Environmental Au, 2024, 4(6): 271-291.

[5] Song J, Yu Y, Han X, Yang W, Pan W, Jian S, Duan G, Jiang S, Hu J, Novel MOF (Zr)-on-MOF (Ce) adsorbent for elimination of excess fluoride from aqueous solution[J]. Journal of Hazardous Materials, 2024, 463: 132843.

[6] Liu R, Song J, Zhang Z, Ji L, Yang W, Zhao J, Jian S, Hu J, Ma J, Needle-like PVP@ Ce/Zr-MOFs for the highly efficient selective of fluoride and phosphate from aqueous solution[J]. Separation Purification Technology, 2025: 133267.

[7] Diwan V, Sar S K, Biswas S, Lalwani R, Adsorptive extraction of uranium (VI) from aqueous phase by dolomite[J]. Groundwater for sustainable Development, 2020, 11: 100424.
